# Resident Students at the Buffalo Hospital of the Sisters of Charity

**Published:** 1852-10

**Authors:** 


*Resident Students at the Buffalo Hospital of the Sisters of Charity.*—We are requested to give notice that on the first of November next, two resident students at the Buffalo Hospital of the Sisters of Charity are to be appointed, one for a term of six months, and the other for a year. The students are required to pay to the hospital two dollars per week for board. They must have studied medicine for two years, and attended one course of medical lectures, to be eligible. The situation offers a fine opportunity for acquiring practical knowledge of medicine and surgery. Applications *in the hand writing of the applicants*, with testimonials, may be addressed to Dr. E. Wallis, Buffalo, chairman of a committee to select suitable candidates.

